# A novel brain-controlled wheelchair combined with computer vision and augmented reality

**DOI:** 10.1186/s12938-022-01020-8

**Published:** 2022-07-26

**Authors:** Kaixuan Liu, Yang Yu, Yadong Liu, Jingsheng Tang, Xinbin Liang, Xingxing Chu, Zongtan Zhou

**Affiliations:** College of Intelligence Science and Technology, National University of Defense Technology, Changsha, 410073 Hunan China

**Keywords:** Brain-controlled wheelchair (BCW), Computer vision (CV), Augmented reality (AR), Disability assistance, And Electroencephalogram (EEG)

## Abstract

**Background:**

Brain-controlled wheelchairs (BCWs) are important applications of brain–computer interfaces (BCIs). Currently, most BCWs are semiautomatic. When users want to reach a target of interest in their immediate environment, this semiautomatic interaction strategy is slow.

**Methods:**

To this end, we combined computer vision (CV) and augmented reality (AR) with a BCW and proposed the CVAR-BCW: a BCW with a novel automatic interaction strategy. The proposed CVAR-BCW uses a translucent head-mounted display (HMD) as the user interface, uses CV to automatically detect environments, and shows the detected targets through AR technology. Once a user has chosen a target, the CVAR-BCW can automatically navigate to it. For a few scenarios, the semiautomatic strategy might be useful. We integrated a semiautomatic interaction framework into the CVAR-BCW. The user can switch between the automatic and semiautomatic strategies.

**Results:**

We recruited 20 non-disabled subjects for this study and used the accuracy, information transfer rate (ITR), and average time required for the CVAR-BCW to reach each designated target as performance metrics. The experimental results showed that our CVAR-BCW performed well in indoor environments: the average accuracies across all subjects were 83.6% (automatic) and 84.1% (semiautomatic), the average ITRs were 8.2 bits/min (automatic) and 8.3 bits/min (semiautomatic), the average times required to reach a target were 42.4 s (automatic) and 93.4 s (semiautomatic), and the average workloads and degrees of fatigue for the two strategies were both approximately 20.

**Conclusions:**

Our CVAR-BCW provides a user-centric interaction approach and a good framework for integrating more advanced artificial intelligence technologies, which may be useful in the field of disability assistance.

## Background

Brain-computer interfaces (BCIs), as tools that directly connect users’ brains and external devices, are widely used in education [1, 2], entertainment [3, 4], and clinical disability assistance research [5–7]. Based on BCIs, researchers have developed many interesting applications, one of which is the brain-controlled wheelchair (BCW) [8–10]. Due to their noninvasive nature, relatively high time resolutions, and low costs, electroencephalogram (EEG) signals have been used in many BCW studies [10–12]. An EEG-based BCW collects EEG signals from the user's scalp and decodes user commands from the collected signals. Currently, EEG signals mainly include P300 signal [10, 12], steady-state visually evoked potential (SSVEP) [11], and sensorimotor rhythms (SMRs) [6]. According to the EEG signals used, current BCWs mainly include the P300-based BCWs [11, 12], the SSVEP-based BCWs [11, 13], and the SMRs-based BCWs [14, 15]. A P300-based BCW has relatively high accuracy and a high information transfer rate (ITR), tends not to elicit fatigue and is easy to use [12]. In this study, we developed a P300-based BCW. We used visual stimuli to evoke the P300 signals. Row–column paradigm and single-character (SC) paradigm are two widely used visual stimulation paradigms. The number of options provided by P300-based BCWs is usually small (< = 10), and relevant BCW studies often use the SC paradigm [16]. In this study, we used the SC visual stimulation paradigm to develop our BCW system. We used five electrodes (FC1, FC2, CP1, CP2, and Cz) to collect P300 signals for each user.

Since Tanaka et al. [8] proposed the first EEG-based BCW paradigm, many BCW systems have been developed. Currently, BCWs can be categorized as automatic and semiautomatic. Semiautomatic BCWs refer to BCWs without navigation abilities, and they are typically used to help users move in indoor environments. Users control semiautomatic BCWs by directly sending steering commands. For example, Wang et al. developed a semiautomatic BCW based on P300 signal, which had seven movement options: forward, backward, left, right, acceleration, cancellation, and stopping [17]. When a user wants to move forward, he or she needs to select the option "forward" in the user interface, and then the BCW will move forward, and so forth for other movement options. Relevant studies have suggested that SSVEP-based BCIs [18] and SMRs-based BCIs [6] can be used in semiautomatic BCWs. Yu et al. proposed an asynchronous BCW based on sequential motor imagery. By sequentially imagining the movements of the left and right hands, this BCW could provide six options for its users [6]. A recent study by Li et al. developed a semiautomatic BCW controlled by a hybrid BCI (P300 signal and SSVEP) [11]. Automatic BCWs refer to BCWs with automatic navigation abilities. After a user selects a target, the automatic BCW will automatically reach it. Currently, several navigation strategies are available. For example, Rebsamen et al. realized the navigation function by predefining a path between each candidate target and the BCW. After a user selects a candidate target, the automatic BCW automatically reaches the target along the predefined path [19]. Lopes et al. [20] and Zhang et al. [21] used another navigation strategy: constructing environment maps for their BCWs in advance. After the user selects a target, the automatic navigation algorithms embedded in their BCWs plan a path according to the environment map and guide the BCWs to the target.

Although the progress achieved by the existing BCW studies is encouraging, we can still improve the performance of current BCWs. In our study, we intend to improve the target selection speeds of current BCWs and improve the practicability of BCWs in unfamiliar environments, where the environmental information cannot be calibrated in advance. Computer vision (CV) can be used in various environments and can detect many categories of objects [22, 23]. Combining CV with BCWs may improve the BCW performance [9, 10]. To this end, in this study, we propose a BCW combined with CV and augmented reality (AR) technology or CVAR-BCW. Our CVAR-BCW uses a CV module to automatically detect objects in the immediate environment and encodes the detected objects as BCW options. When the user wants to select a target, they can simply choose the target of interest in the user interface, and then the CV module will automatically guide our BCW to the target. In the CV research field, compared with the two-stage CV algorithms, the YOLOv3 [24] algorithm has relatively high mean average precision and relatively high frames per second. Therefore, our CVAR-BCW uses the YOLOv3 algorithm.

Traditional BCWs use computer screens to display their user interfaces [8–10]; this approach has two major disadvantages: (1) computer screens are not wearable, and (2) the fields of view of computer screens are relatively small. When a user selects BCW options on a computer screen, they cannot simultaneously observe the current environment. If some obstacles are present in the environment and the user does not observe them, traditional BCWs may not be safe [25]. Using a translucent head-mounted display (HMD) can well address these issues. For our CVAR-BCW, we use an HMD and a video see-through AR technique [26, 27] to build an immersive user interface, which simultaneously shows the real environment and the virtual stimuli employed to evoke the user's P300 signal.

Our CVAR-BCW provides an end-to-end interaction strategy. Compared with current semiautomatic BCWs, the proposed CVAR-BCW can reach the targets selected by users faster. Our CVAR-BCW system does not require hard-coded environmental information, since the CV module can detect environmental information in real time, making our system usable in unfamiliar environments. Due to the integration of AR technology, compared with BCWs based on computer screens, our CVAR-BCW can display environmental information in a more intuitive and immersive way. In addition, our user interface is wearable.

The degrees of fatigue and the workloads of users are important factors in the research and development of BCWs. Relevant studies [28–31] often use the National Aeronautics and Space Administration-Task Load Index (NASA-TLX) [32] to measure user workload and the Fatigue Questionnaire proposed by Trudie et al. [33] to measure user fatigue. In our study, after completing the experiments, each user was asked to complete the NASA-TLX and the Fatigue Questionnaire. The NASA-TLX includes six subscales: mental demand, physical demand, temporal demand, performance, effort and frustration. The full score of each subscale is 100, and the total score of NASA-TLX is the average score of all subscales. The higher the score, the greater the workload. For example, if a user X scores 100 and another user Y scores 50, the workload of user X is greater than that of user Y. The full score of the Fatigue Questionnaire is 100. The questionnaire includes 14 items, and each item is approximately seven marks. Similarly, a high score on the Fatigue Questionnaire indicates a higher degree of fatigue.

In the rest of this paper, the Methods section describes the subjects, the structure and principle of the proposed CVAR-BCW, the experimental procedure, the EEG collection and processing algorithm, and the utilized performance metrics. The Results section describes the experimental results. The Discussion section discusses the significance and limitations of this study, as well as future work ideas. The Conclusion section concludes this study.

## Results

### P300 signals

**Fig. 1:**
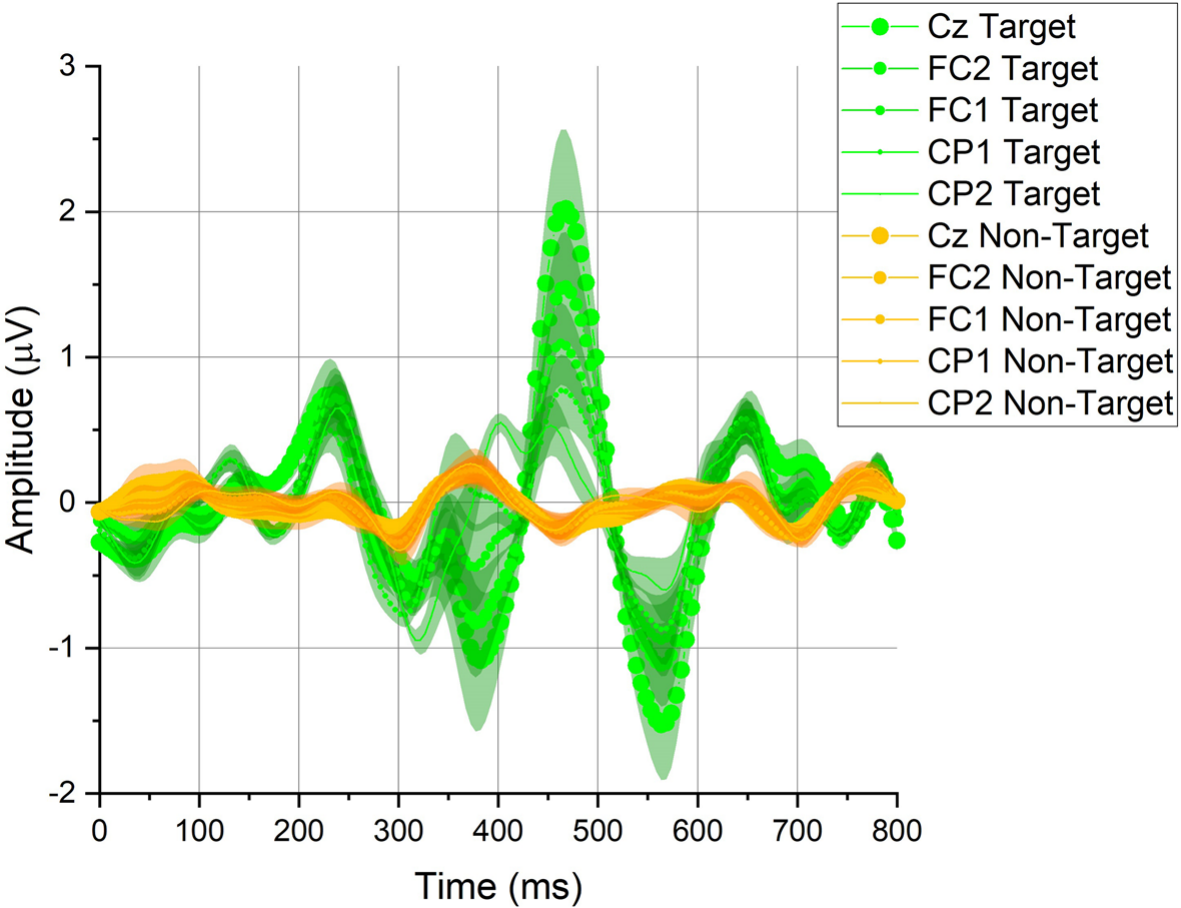
The signals recorded from each EEG channel. The green lines are the average target signals of all subjects, and the yellow lines are the average non-target signals of all subjects. The shaded areas are the corresponding error bands

**Fig. 2:**
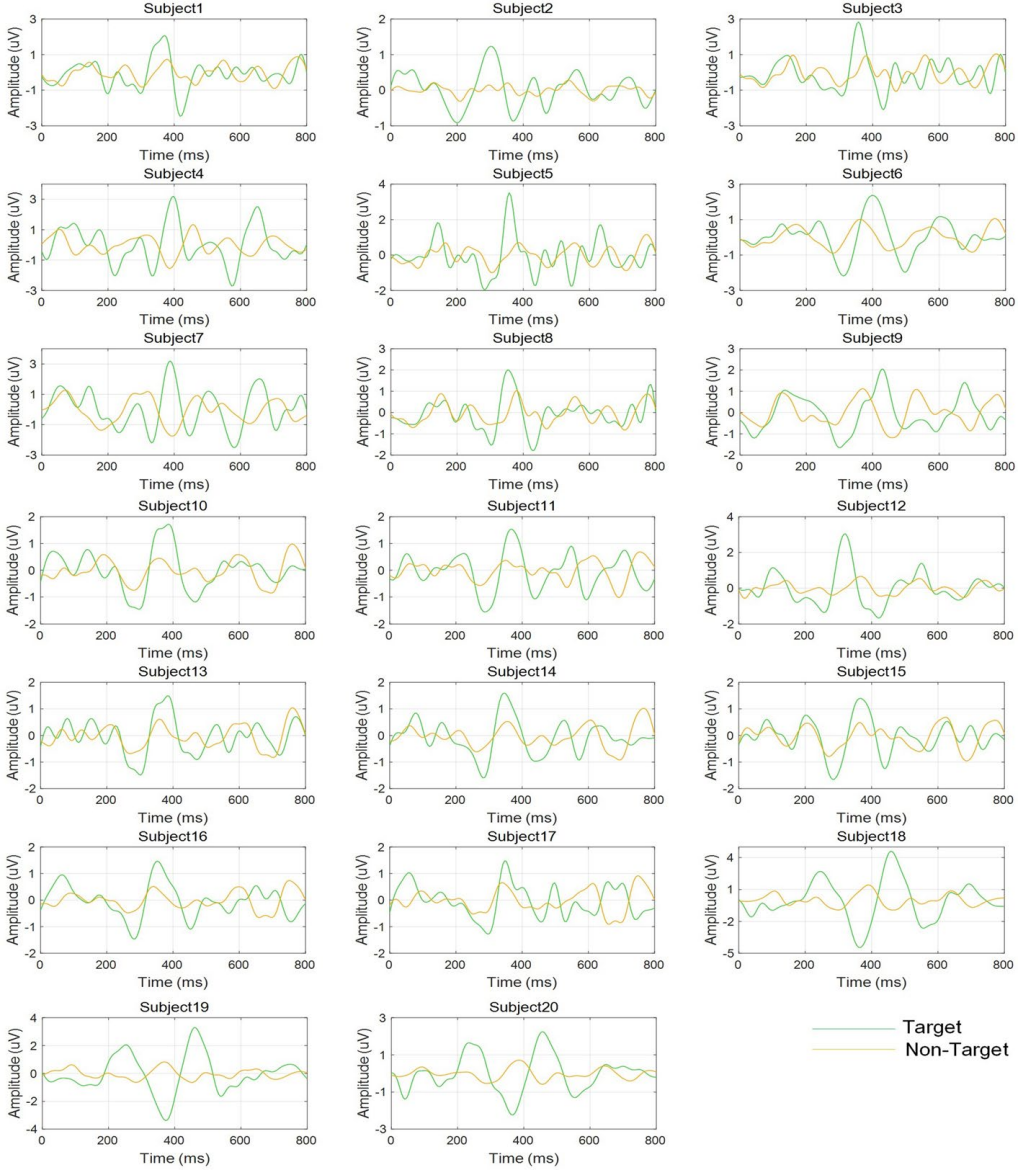
The target and non-target signals of each subject

We collected EEG signals from five electrodes (FC1, FC2, CP1, CP2, and CZ). According to the collected EEG signals, we calculated the average signals and the corresponding error bands for each EEG channel; we showed them in Fig. 1. For the target signals, after the stimuli appeared for approximately 400 ms, obvious P300 components were observed in all channels, and the largest P300 amplitude appeared in Cz (approximately 2 μV). The non-target signals were relatively small, and the amplitudes were all less than 0.5 μV. Figure 2 shows the target signals and non-target signals of all subjects. Despite individual differences, obvious P300 components could be observed in the target signal of each subject (between 200 and 500 ms, the amplitudes were all approximately 1–3 μV). Similar to Fig. 1, the amplitudes of all non-target signals were much smaller than those of the target signals (all less than 1 μV).

### BCW performance

We generated Table 1 according to the performance of all subjects in the online experiment. The average accuracies and average ITRs for the subjects in the semiautomatic mode and automatic mode were almost the same: 84.1% (the maximum and minimum values were 95.2% and 75.8%, respectively) and 8.3 bits/min (the maximum and minimum values were 11.4 bits/min and 6.3 bits/min, respectively) for the semiautomatic mode and 83.6% (the maximum and minimum values were 100% and 71.4%, respectively) and 8.2 bits/min (the maximum and minimum values were 13.4 bits/min and 5.5 bits/min, respectively) for the automatic mode. However, the average time and average number of commands taken by the CVAR-BCW to reach each target in the two modes were different. In the semiautomatic mode, the average time spent was approximately 93.4 s (the maximum and minimum values were 120 s and 78 s, respectively), and the average number of commands given was approximately 2.7 (the maximum and minimum values were 3.5 and 2.1, respectively); in the automatic mode, the average time spent was approximately 42.4 s (the maximum and minimum values were 54 s and 36 s, respectively), and the average number of commands given was approximately 1.2 (the maximum and minimum values were 1.4 and 1.0, respectively). Intuitively, the automatic mode was faster than the semiautomatic mode. We used SPSS (version 21.0), which is widely used in statistics, to analyze the data shown in Table 1. The results showed that at the significance level of 0.05, the accuracy, ITR, average time and average number of commands recorded in each of the two modes did not follow a normal distribution. We could not use a paired t test, so in this study, we used the Mann–Whitney U test. The results showed that there were no significant differences in the accuracy (the statistical value was −0.380, the p value was 0.718, and the effect size was −0.165) and ITR (the statistical value was −0.339, the p value was 0.738, and the effect size was −0.003) between the two modes at the level of 0.001, while the average time (the statistical value was −5.442, the p value was 0.000, and the effect size was −5.338) and average number of commands (the statistical value was −5.436, the p value was 0.000, and the effect size was −5.324) in the automatic mode were significantly smaller than those in the semiautomatic mode.

**Table 1:** Performance overview of the CVAR-BCW

Subject	Age (years)	Sex	Semiautomatic mode				Automatic mode			
			Accuracy (%)	ITR (bits/min)	Average time (s)	Average commands	Accuracy (%)	ITR (bits/min)	Average time (s)	Average commands
S1	29	M	84.0	8.1	90.0	2.5	83.3	8.0	42.0	1.2
S2	27	M	82.6	7.8	84.0	2.3	90.9	10.0	39.0	1.1
S3	25	F	82.1	7.7	102.0	2.8	76.9	6.6	48.0	1.3
S4	29	M	79.3	7.1	90.0	2.9	71.4	5.5	48.0	1.4
S5	31	M	95.2	11.4	78.0	2.1	100	13.4	36.0	1.0
S6	25	M	88.5	9.3	84.0	2.6	90.9	10.0	38.0	1.1
S7	30	F	84.0	8.2	90.0	2.5	90.9	10.0	39.0	1.1
S8	33	M	80.7	7.4	108.0	3.1	71.4	5.5	48.0	1.4
S9	24	M	84.6	8.3	96.0	2.6	71.4	5.5	42.0	1.4
S10	26	M	91.3	10.1	80.0	2.3	100	13.4	36.0	1.0
S11	25	M	88.9	9.4	96.0	2.7	83.3	8.0	42.0	1.2
S12	34	F	87.0	8.9	78.0	2.3	90.9	10.0	38.0	1.1
S13	29	F	77.1	6.6	120.0	3.5	71.4	5.5	54.0	1.4
S14	29	M	81.5	7.6	84.0	2.7	90.9	10.0	39.0	1.1
S15	28	M	85.7	8.6	96.0	2.8	83.3	8.0	42.0	1.2
S16	22	M	80.0	7.2	108.0	3.0	71.4	5.5	49.0	1.4
S17	25	M	75.8	6.3	114.0	3.3	71.4	5.5	45.0	1.4
S18	24	F	79.3	7.1	108.0	2.9	76.9	6.7	42.0	1.3
S19	29	F	87.0	8.9	78.0	2.3	90.9	10.0	39.0	1.1
S20	26	F	88.0	9.2	84.0	2.5	83.3	8.0	42.0	1.2
AVG ± STD	27.5 ± 3.1	––	84.1 ± 5.0	8.3 ± 1.2	93.4 ± 13.0*	2.7 ± 0.4*	83.6 ± 9.8	8.2 ± 2.5	42.4 ± 4.8*	1.2 ± 0.1*

F denotes female and M denotes male

^*^p value < 0.001 with Mann–Whitney U tests, “––” means not applicable

**Fig. 3:**
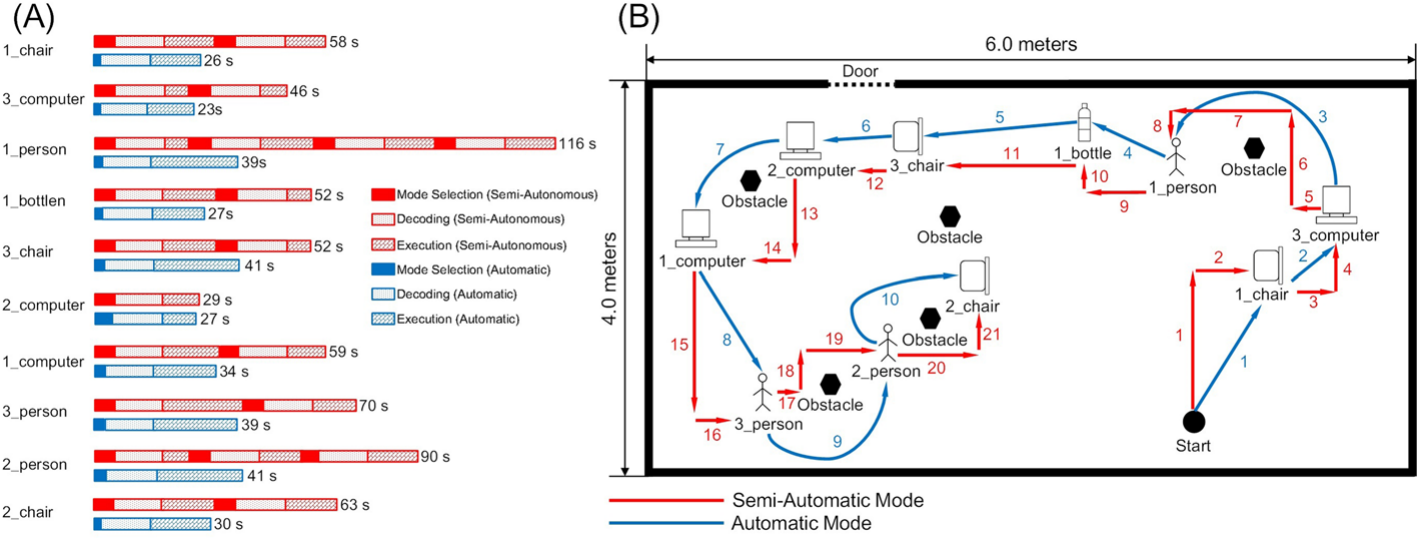
The motion of a subject X in the online experiment. **A** Shows the motion trajectory, and **B** shows the time cost to reach each target. There were three persons, three chairs, three computers, one bottle, and several obstacles in the room. To distinguish objects belonging to the same categories, we added a number before each object, e.g., 1_person was the first person in the room

In Fig. 3, we showed the motion trajectory of subject X and the time spent reaching each target. The experiment was conducted in a room with a size of 4.0 m × 6.0 m. The first target chosen by subject X was the chair (i.e., labeled as 1_chair on the interface). In the automatic mode, the CVAR-BCW directly reached the target, and the time to reach the target was 26 s. In the semiautomatic mode, the CVAR-BCW could not directly reach the target because the semiautomatic mode did not have navigation capability. The CVAR-BCW first moved forward and then translated left. The total time spent in semiautomatic mode was 58 s. The second target chosen by subject X was the computer (i.e., labeled as 3_computer on the interface). In the automatic mode, the CVAR-BCW could directly reach the target, and the time spent (23 s) by subject X was less than that (46 s) in the semiautomatic mode. An obstacle was located between the second target and the third target. In the automatic mode, the CVAR-BCW directly reached the third target person (i.e., labeled as 1_person on the interface) within 39 s. In the semiautomatic mode, the CVAR-BCW did not have navigation capability and could not directly reach this target. Subject X had to plan an appropriate path and move the CVAR-BCW along the path to reach the target, which took the subject 116 s. For the remaining targets, the motion trajectories in the semiautomatic mode were similar to those in automatic mode. However, due to the lack of navigation capability, the semiautomatic mode required more time.

### Workload and degree of fatigue

Table 2 shows the scores yielded by each subject on the NASA-TLX and the Fatigue Questionnaire. Although there were some individual differences, the average scores that the subjects received were relatively low compared with the full scores of 100. The average scores of mental demand, physical demand, temporal demand, performance, effort, frustration, fatigue were 23.5, 22.3, 24.8, 22.3, 21.5, 17.5, and 20.8, respectively. According to relevant studies [32, 33], the workload and degree of fatigue of our CVAR-BCW were relatively low.

**Table 2:** Scores of the NASA-TLX and the Fatigue Questionnaire

Subject	Mental demand	Physical demand	Temporal demand	Performance	Effort	Frustration	Fatigue
S1	25	50	50	40	40	25	36
S2	25	5	45	45	50	45	36
S3	20	30	30	25	5	15	21
S4	50	35	30	30	25	20	29
S5	25	20	40	45	20	20	14
S6	25	20	20	55	25	15	29
S7	15	15	10	5	5	10	7
S8	15	15	25	25	20	20	14
S9	10	5	20	25	20	15	21
S10	5	20	15	5	25	5	7
S11	15	20	10	15	20	10	14
S12	40	35	5	5	40	5	21
S13	45	20	30	10	5	5	21
S14	20	25	15	10	25	5	21
S15	30	5	40	10	5	50	48
S16	15	15	20	25	25	5	7
S17	40	35	20	10	10	10	14
S18	15	15	25	5	25	20	14
S19	15	25	20	30	20	20	21
S20	20	35	25	25	20	30	21
AVG ± STD	23.5 ± 12.0	22.3 ± 11.8	24.8 ± 12.0	22.3 ± 15.3	21.5 ± 12.2	17.5 ± 12.6	20.8 ± 10.6

### Effect of the number of flashes

**Fig. 4:**
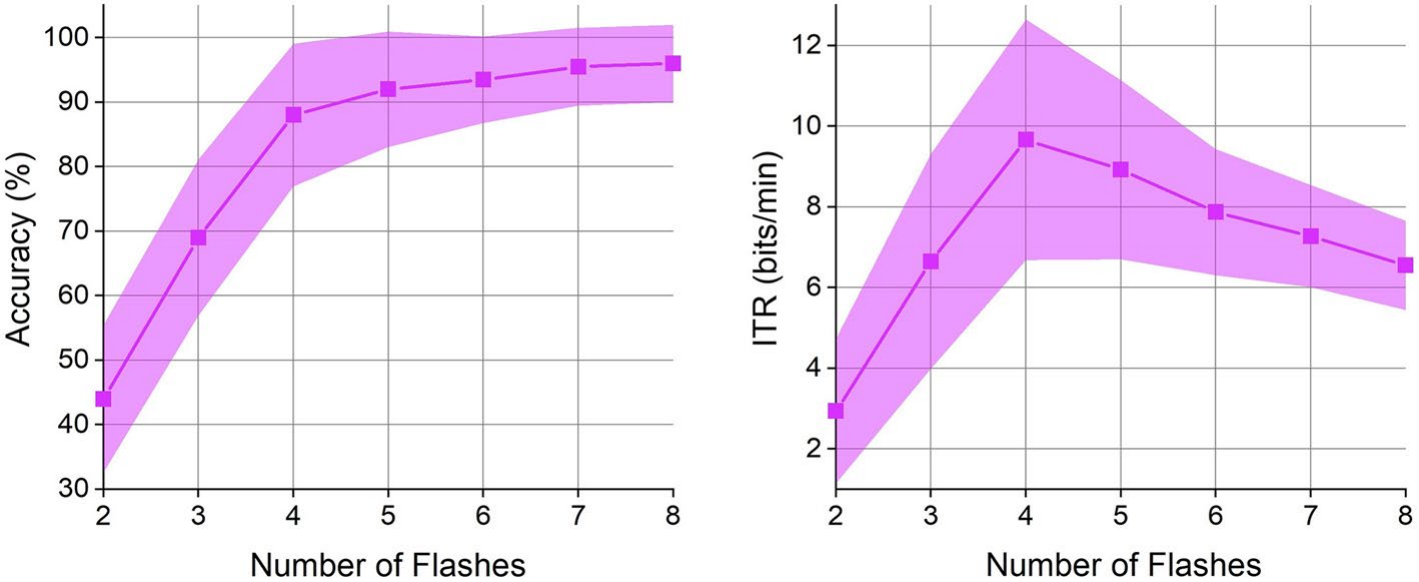
The effect of the number of flashes on the accuracy and ITR

Using the data collected from the optimization experiment, we plotted Fig. 4. When the number of flashes was small, increasing the number of flashes quickly improved the decoding accuracy and the ITR since more flashes provided more P300 features to the classifier. However, when the number of flashes was larger than an optimal value, the classifier reached its limit, and the decoding accuracy gradually stabilized. More flashes cost more time, decreasing the ITR. According to the experimental results in Fig. 4, when there were four flashes, the ITR reached the maximum value of approximately 10 bits/min. In this study, we chose four flashes in the online experiment.

### Errors occurring in automatic mode

In the online experiment, once the CVAR-BCW failed to reach a target, we orally asked the subjects about the possible reasons for this phenomenon and recorded them. In the semiautomatic mode, the CV module was turned off. If the CVAR-BCW did not reach a target, the reason was usually a BCI decoding error, so we did not analyze the errors that occurred in the semiautomatic mode. In the automatic mode, the possible errors were more complicated. We divided the number of each error type by the total number of all errors to obtain the percentage of each error type and plotted the following pie chart. In our experiment, the largest error that occurred was the decoding error (approximately 48%), followed by the navigation error (approximately 28%). The percentages of these two errors were larger than those of the remaining errors.

## Discussion

In the automatic mode, the P300 decoding algorithm and the number of options were the same as those in the semiautomatic mode, so the accuracies and ITRs between the two modes were almost the same. In the automatic mode, after the user selected a target, the wheelchair could automatically reach the target. However, in the semiautomatic mode, if the user wanted to reach a target, the user needed to continuously send steering commands to control the BCW, e.g., when to move forward and when to move left. The wheelchair control strategy in the automatic mode was simpler than that in the semiautomatic mode, so it took less time and fewer commands to reach the target in the automatic mode.

Currently, there are two categories of BCWs: semiautomatic and automatic. Users directly employ steering commands to control semiautomatic BCWs [17, 34]. For example, a recent study by Yu et al. developed a semiautomatic BCW with 11 movement options. The user controls the BCW to move forward, stop, accelerate and decelerate through P300 signal and SMRs [34]. Automatic BCWs usually have navigation modules. The control strategy for automatic BCWs is different from that of semiautomatic BCWs, and the user controls an automatic BCW by selecting the target of interest [19–21]. For example, Rebsamen et al. developed a BCW for indoor environments in which the positions of all objects were calibrated in advance [19]. The user could select a target in the user interface, and then the wheelchair would automatically reach the target along the calibrated path. The automatic BCW developed by Zhang et al. can construct a global environmental map in advance. After the user selects a target, the navigation module embedded in their BCW plans a path and guides the BCW to the target. The above studies improved the practicability of BCWs and enriched their applications. Our CVAR-BCW can be regarded as an extension of existing relevant studies. The advantage of our CVAR-BCW is that we used CV to automatically detect and encode objects in the environment, and the BCW could show the detected objects to users through an end-to-end interaction strategy. After the user selected a target, the proposed CVAR-BCW could directly reach the target. The users did not need to frequently send steering commands. The target selection speed of our CVAR-BCW was faster than that of semiautomatic BCWs, and our system reduced the user's workload to some extent. Compared with automatic BCWs, our CVAR-BCW did not require an environment map and could be better used in unfamiliar environments. In addition, our CVAR-BCW has an automatic mode and a semiautomatic mode. Users can select the appropriate mode according to their current environment. If the user wants to quickly reach a target, e.g., to grasp a bottle of water, he or she can choose the automatic mode; if the user wants the BCW to help them move in a room, e.g., move forward several meters, the user can choose the semiautomatic mode, which is useful for users with impaired motor function.

Currently, there are many BCW studies [6, 21] and robotic arm studies [35, 36]; however, few studies combine both topics. A BCW combined with a robotic arm is useful for many scenarios. For example, if a paraplegic user wants to drink water, the robotic arm installed on the BCW can grasp a bottle of water and help the user drink. The combination of BCWs and robotic arms can also promote the interdisciplinary development of relevant studies. In this study, we installed a multidegree-of-freedom robotic arm on our CVAR-BCW.

Current BCWs typically use computers screen to build their user interfaces [17, 19, 21, 34]. Paraplegic patients might be uncomfortable sitting beside a computer screen for a long time [37]. In this study, our CVAR-BCW used an HMD as the user interface and AR technology to display information. Theoretically, a patient can see the surrounding environment from the first-person perspective and select targets through the HMD in any place. When the patient is lying in bed, he or she can even remotely control the BCW to bring him or her a bottle of water. Our system provided paraplegic users with a more user-centric interaction strategy and a good framework that can be integrated with many interesting technologies, such as the metaverse [38, 39] and teleoperation approaches [40–42].

The proposed CVAR-BCW has some limitations; the performance of the developed CVAR-BCW may be affected by light since the CV module we used cannot accurately detect objects in a dark environment. In addition, although six options are suitable for various tasks, users may need more BCW options for some complex scenarios. Although our CVAR-BCW can automatically reach the target selected by the user, the chosen path might not be optimal. We need to integrate a more advanced navigation framework into our CVAR-BCW. We did not measure the influence of the learning effect on our results. In semiautomatic mode, error types (1) and (2) were usually caused by the misoperation of users and might not be related to the CVAR-BCW, and we did not calculate error types (1) and (2). In semiautomatic mode, error types (3) and (5) were not observed. In semiautomatic mode, if the CVAR-BCW did not reach the target, error type (4) was the main reason. In semiautomatic mode, error type (6) might occur, but we did not analyze this error type, which is a limitation of our study.

**Fig. 5:**
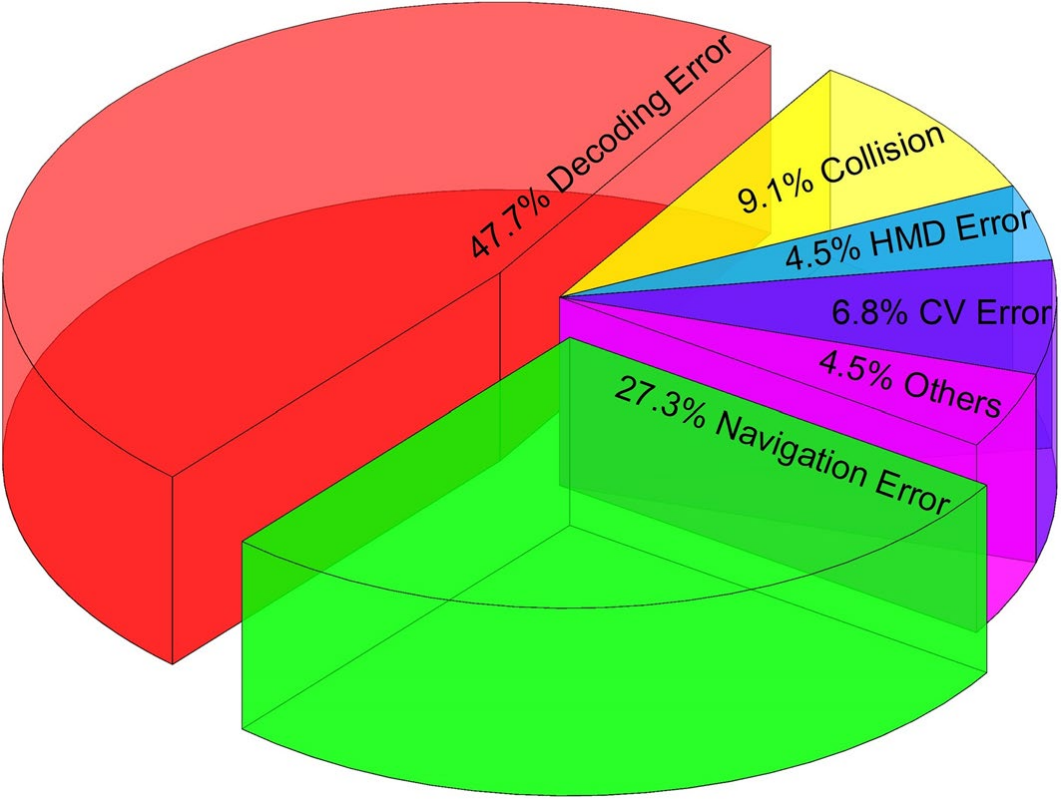
The error types recorded in the automatic mode

In automatic mode, we analyzed possible error types caused by combining CV and AR with BCWs. As shown in Fig. 5, most errors were navigation and decoding errors. In the future, we will use more advanced P300 decoding algorithms, such as a Bayesian linear discriminator [43], to develop the classifier. In addition, Fig. 4 shows that increasing the number of flashes can improve the accuracy of our approach to some extent. In future work, we will use a dynamically changeable number of flashes: for users with high accuracy, we will use fewer flashes; for users with low accuracy, we will use more flashes. The study conducted by Yin et al. [44] indicated that a hybrid BCI may have better performance than a traditional P300-based BCI. Li et al. developed a BCW controlled by a hybrid BCI (combining a P300 signal with an SSVEP signal) [11]. Their experimental results suggested that the hybrid BCI improved the accuracy and response time of the wheelchair. In the future, we will modify the CVAR-BCW based on hybrid BCIs. In the future, we can put an SSVEP stimulus and a P300 stimulus on each option to simultaneously evoke the user's SSVEP signal and P300 signal and then decode the target from these P300 and SSVEP signals. The simultaneous localization and mapping (SLAM) algorithm can be used in unfamiliar environments where information cannot be calibrated in advance. The A* algorithm is a commonly used navigation algorithm. We intend to improve the performance of our CVAR-BCW in unfamiliar environments. In the future, we can use a visual SLAM framework based on a Kinect sensor to construct a global environment map and then use the A* algorithm to navigate our wheelchair to the targets of interest [21].

## Conclusion

In this study, we developed the CVAR-BCW, a BCW combined with CV and AR. Our CVAR-BCW had two modes: automatic and semiautomatic. In the automatic mode, the CVAR-BCW could automatically detect the current environment and navigate to targets selected by the user. In the semiautomatic mode, users could control each movement of the CVAR-BCW. The experimental results showed that our CVAR-BCW performed well in indoor environments, and the workload and degree of fatigue of users were low. In the automatic mode, the average accuracy was 83.6%, the average ITR was 8.2 bits/min, and the average time required to reach one target was 42.4 s. In the semiautomatic mode, the average accuracy, ITR, and time to reach one target were 84.1%, 8.3 bits/min, and 93.4 s, respectively. Our CVAR-BCW may be useful for disability assistance and provides a possible direction for the development of user-centric BCWs.

## Methods

### Subjects

This study was approved by the Ethics Committee of Xiangya Hospital, Central South University. All processes of the study were in line with the 1964 Declaration of Helsinki. All subjects provided written consent before the experiment. This study recruited 20 healthy subjects with normal vision or normal vision after correction (ranging from 24 to 33 years old, 7 females and 13 males). Relevant studies have shown that drinking stimulating beverages (such as coffee), staying up late, and sleeping for too short a period can affect people's cognition and brain activity [45]. To reduce the influences of these factors on our study, we asked the subjects recruited not to drink stimulating beverages, not to stay up late, and to sleep more than eight hours before the experiment. The experiment was conducted in a bright room that was approximately 4.0 m × 6.0 m in size. During the experiment, all doors and windows were closed to maintain silence (minimum 40.2 dB, maximum 52.9 dB, mean 46.4 dB), and no one was allowed to stay in the room except the researchers and subjects. Before the experiment, we illustrated the experimental procedure and how to use our CVAR-BCW in detail. Subjects could try the CVAR-BCW until they were completely familiar with it.

### System architecture

**Fig. 6:**
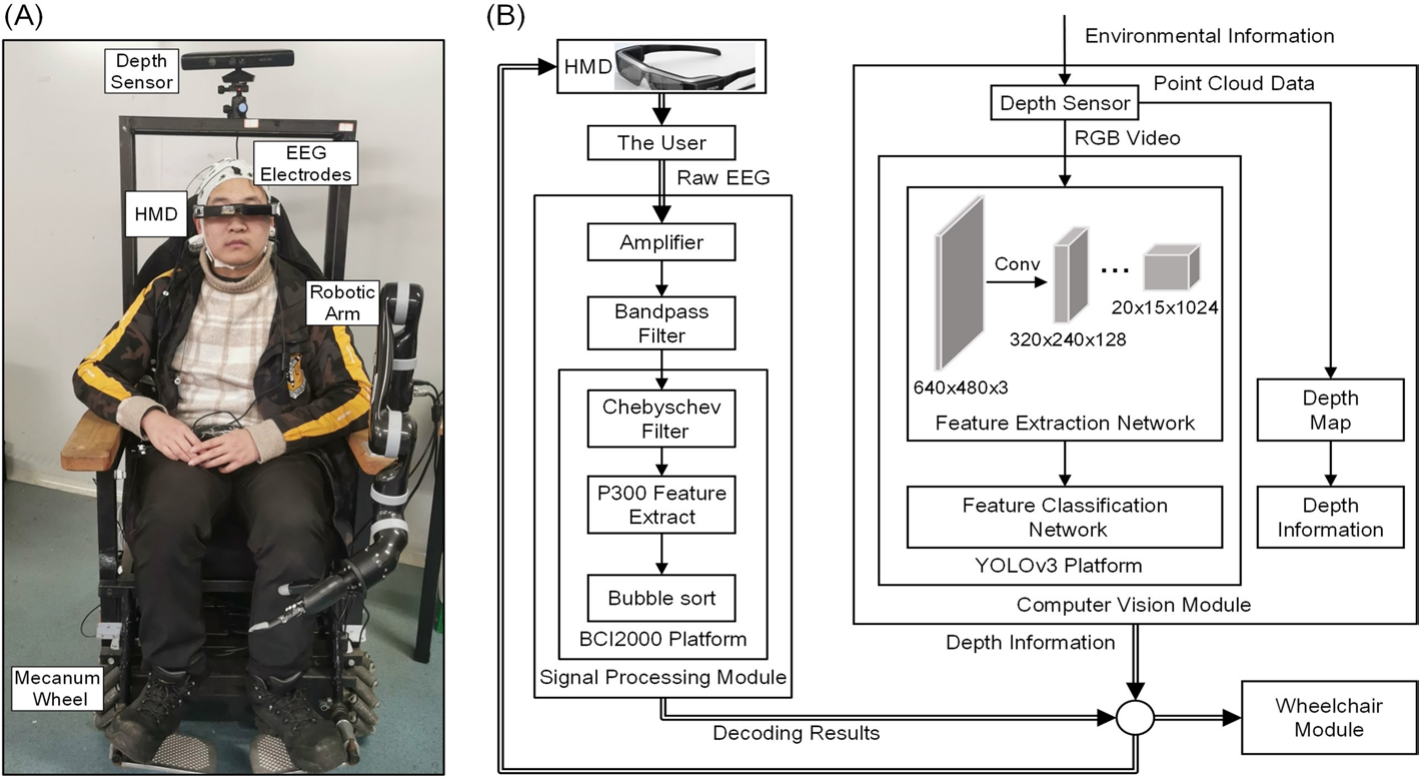
The actual picture and architecture of our CVAR-BCW. **A** is the actual picture, and **B** is the system architecture

**Fig. 7:**
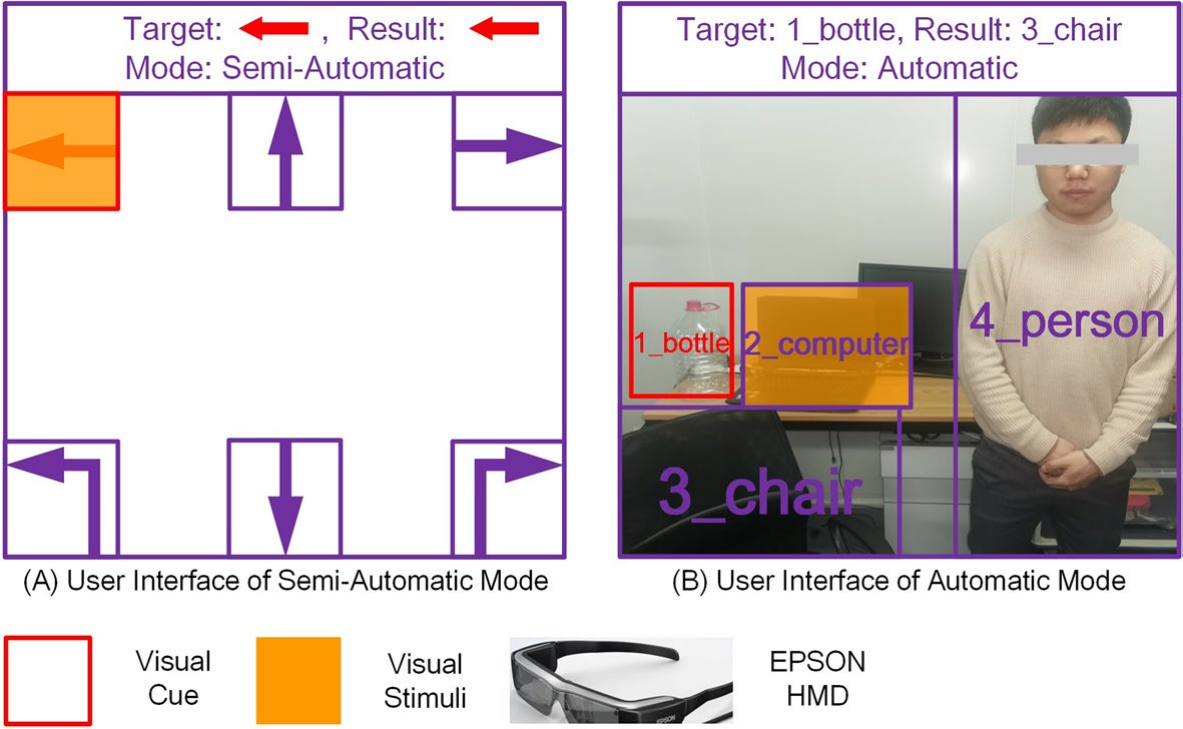
User interfaces for automatic mode and semiautomatic mode. The CVAR-BCW is developed for indoor environments. The color of the BCW options and the color of stimuli should be different from most objects in indoor environments to reduce visual interference. In indoor environments, there are relatively few red, purple, and orange objects. Therefore, we set the target option, the non-target options, and the stimuli of our BCW to red, purple, and orange, respectively. The orange stimuli were set to be translucent so that users could simultaneously see stimuli and the environment. At the top of the user interfaces, the system automatically displayed the current target (after the text "Target"), the current decoding result (after the text "Result"), and the current BCW mode (after the text "Mode")

Figure 6 shows the system architecture and an actual picture of our CVAR-BCW. The length, width, and height of our BCW were approximately 1.0 m, 1.1 m, and 1.5 m, respectively. The BCW consisted of four modules: a signal processing module, a computer vision (CV) module, a user interface, and a wheelchair module. We used a local area network and a nonblocking user datagram protocol for communication between the modules:

- In the signal processing module, we first used Ag/AgCl electrodes to collect EEG signals from the user's scalp. Then, we used an actiCHamp amplifier (Brain Products, Germany) and a bandpass filter to preprocess the collected raw EEG signals. Next, a BCI2000 platform [46] was used to decode the user commands from the preprocessed signals.
- In the CV module, we used a Kinect Xbox 360 depth sensor (Microsoft, USA) to collect environmental information in real time. The depth sensor could simultaneously output the RGB video captured in the current environment and the point cloud data of all objects in the current environment. We used a Darknet-53 framework-based YOLOv3 platform to detect environmental objects in the captured RGB video [24]. Then, the CV module automatically encoded the detected environmental objects as the BCW options. The CV module processed the point cloud data in real time to obtain a depth map of the current environment. Next, in the depth map, the CV module used a depth measurement algorithm to measure the depth information of each object (e.g., the distance from each object to the CVAR-BCW). The depth information was sent to the wheelchair module.
- We used an EPSON BT350 HMD (Seiko Epson Corporation, Japan) to develop the user interface. The user interface showed the BCW options (from the CV module), the visual stimuli used to evoke the user's P300 response, and the decoding results (from the signal processing module). Figure 7 shows an example of the user interface.
- The purpose of this study is to provide a BCW that can work stably in indoor environments. Relevant BCW studies have suggested that a mecanum wheel can translate and rotate in any direction [47] and is thus suitable for indoor environments [6]. A Kinova robotic arm (Kinova Robotics, Canada) can flexibly grasp many objects in our daily life, e.g., bottles and cups [48], which may be useful in indoor scenarios. The wheelchair module of the CVAR-BCW consisted of a chassis with four mecanum wheels and a Kinova robotic arm installed on the wheelchair. When the user selected a target, the wheelchair module would immediately read the decoding result derived from the signal processing module, read the depth information obtained from the CV module, and plan a path to the target. Next, the chassis would move to the target, and the robotic arm would grasp the target.

In indoor environments, BCWs combined with CV provide a fast end-to-end interaction strategy for users. However, when a user only wants to move forward or backward, rather than select a certain target, such BCWs cannot complete the task well. To develop a BCW suitable for various scenarios, our CVAR-BCW contained two modes: an automatic mode and a semiautomatic mode. In automatic mode, the CVAR-BCW uses the CV module to detect and encode environmental objects and can automatically navigate to the targets selected by the user. In semiautomatic mode, the navigation function is turned off, and the user can control each movement of the CVAR-BCW. Similar to traditional BCWs, in semiautomatic mode, the user can decide when the CVAR-BCW moves forward and when the CVAR-BCW moves backward through their P300 signal. We installed a switch on the CVAR-BCW, by which a user can change the mode.

Figure 7A shows the user interface of the semiautomatic mode. There were six options in the user interface: "Forward", "Left Turn", "Left Translation", "Right Turn", "Right Translation", and "Backward". A red visual cue was used to mark the current target, i.e., the current target was "Left Translation". Orange rectangular stimuli were used to elicit the user's P300 response. Figure 7B shows the user interface of the automatic mode. We used the same visual cue and stimuli. In automatic mode, the BCW options were the environmental objects detected by the CV module. The number of environmental objects may not be constant, so the number of BCW options is changeable. In this study, we wanted to keep the number of options in the automatic and semiautomatic modes the same. In semiautomatic mode, the number of options is six. If there were too many objects (> 6) in the current environment, in automatic mode, the user interface would randomly show six of them. If the number of environmental objects was less than six, the user interface would introduce fake options to fill the inadequate options. For example, in Fig. 7B, the user interface included four options from the current environment ("1_bottle", "2_computer", "3_chair", and "4_person") and two fake options. Fake options were set as invisible to prevent distractions. If a fake option was decoded as the current result, the CVAR-BCW would not execute the command because a fake option did not represent any target.

### Wheelchair navigation and robotic arm control strategy

This study aimed to test the advantages of combining CV and AR with traditional BCWs. The wheelchair navigation and robotic arm control strategies used in our CVAR-BCW were relatively simple. In this study, we predefined the current position of the CVAR-BCW as the origin of a Cartesian coordinate system, and the positive directions of the X axis, Y axis and Z axis were horizontal right, vertical down and horizontal forward, respectively. The depth sensor collected the environmental information within four meters in front of the BCW, and then the CV module processed the collected information in real time. After finding a target, the CVAR-BCW would directly go to the target. For example, if the target "person" was three meters away from the CVAR-BCW and was in the left front, the CVAR-BCW would first turn left and then move forward for three meters. If an obstacle was found on the path, the CVAR-BCW would randomly rotate or translate until it avoided the obstacle (this path may not be optimal).

Before conducting experiments, we needed to calibrate the grasping posture of the Kinova robotic arm [49]. For different objects, the grasping postures were different. For each target, we needed to manually operate the robotic arm to grasp the target and then select the end effector of the robotic arm on the depth map. Next, we needed to turn on the CV module. The CV module automatically recorded the coordinates of the selected end effector in the three-dimensional world. According to the recorded coordinates, the algorithm integrated into the robotic arm determined the appropriate grasping posture. In experiments, the robotic arm could only repeat the calibrated posture, which meant that the robotic arm could not grasp moving or uncalibrated objects.

### Experimental procedures

Before the experiment, each subject participating in this study was informed of the experimental procedure and the experimental purpose. Subjects could try the CVAR-BCW several times until they were fully familiar with our system. The experiment included three parts: (a) an offline experiment, (b) an optimization experiment, and (c) an online experiment. After the experiments, each subject was asked to immediately complete the NASA-TLX and the Fatigue Questionnaire so that we could measure the workload and degree of fatigue of each subject. The scores of the NASA-TLX and the Fatigue Questionnaire are shown in Table 2.

**Fig. 8:**
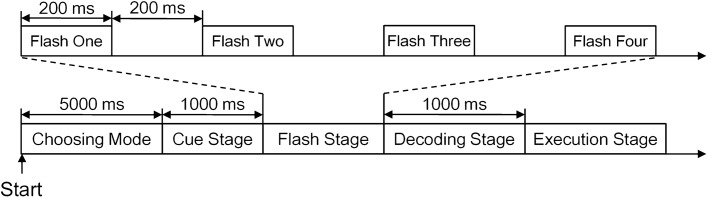
The time course. In the offline experiment, the time course included four stages: the mode selection stage, cue stage, flash stage, and decoding stage. In the flash stage, we used orange rectangular stimuli to construct the flash. Each flash lasted for 400 ms, each stimulus appeared for 200 ms and then disappeared for 200 ms, and four flashes appeared for each option. The parameters used in the optimization experiment were the same as those used in the offline experiment, but the number of flashes in the optimization experiment increased from 2 to 8. The parameters used in the online experiment were the same as those used in the offline experiment, and the flash stage also contained 4 flashes. However, the online experiment included one more stage: the execution stage, in which the BCW executed the decoding results

Figure 8 shows the time course of the experiments. First, a subject was asked to select a mode within 5000 ms. The subjects could select modes through a switch installed on our CVAR-BCW. If the switch was pressed within 5000 ms, the automatic mode would be immediately activated; otherwise, the semiautomatic mode would be activated. At this stage, the HMD only showed the RGB video captured by the depth sensor but did not show the visual cue and stimuli. Next, the HMD showed the corresponding user interface and the visual cue. One thousand milliseconds later, the user interface showed the visual stimuli to evoke the subject’s P300 potential. According to the oddball paradigm [50], we used a stimulus onset asynchrony of 400 ms, i.e., each stimulus appeared for 200 ms and disappeared for another 200 ms. After the flash stage, the BCW decoded user commands within 1000 ms. In the offline experiment and the optimization experiment, our BCW would not execute user commands and would only return the decoding results through the user interface. The time courses of the offline experiment and the optimization experiment included four stages: choosing mode, cue stage, flash stage, and decoding stage. In the online experiment, our CVAR-BCW executed the decoding results, and the time course included an additional execution stage.

#### Offline experiment

In the offline experiment, each subject was asked to use the automatic mode and semiautomatic mode to select 15 targets, respectively. The order of the two modes was not important. For example, a subject could use the automatic mode to select the first 15 targets and then use the semiautomatic mode to select the last 15 targets, or vice versa. In the offline experiment, the number of flashes was four. The purpose of the offline experiment was to collect enough EEG signals to train the classifier [51] needed in the online experiment. After the offline experiment, each subject rested for two minutes.

#### Optimization experiment

The number of flashes might affect the performance of a BCI [44, 52]. In the optimization experiment, we wanted to test the effect of the number of flashes on the CVAR-BCW performance. Each subject was asked to finish seven blocks with ten targets each. The number of flashes increased with the number of blocks: there were two flashes in the first block, three flashes in the second block, and so forth until the last block (eight flashes). For each block, each subject was asked to select five targets from the semiautomatic mode and the other five targets from the automatic mode. The order of the two modes was not important. The visual cue and stimuli used in the optimization experiment were the same as those in the offline experiment.

#### Online experiment

In the semiautomatic mode, when the CVAR-BCW reached the target, subjects needed to press the switch on the BCW to stop it. In the automatic mode, the BCW could automatically stop near the target. In the online experiment, each subject was asked to use the semiautomatic mode and automatic mode to reach 10 designated targets in the room, respectively. The targets were 1_bottle, 1_person, 2_person, 3_person, 1_chair, 2_chair, 3_chair, 1_computer, 2_computer, and 3_computer. To distinguish objects belonging to the same category, we added a number before each object. For example, if there were two persons in a room, we would name them 1_person and 2_person, respectively. In semiautomatic and automatic modes, the subjects had to successfully reach each target. If the subjects did not reach a target, they had to retry until they succeeded. According to each subject’s performance, we generated Table 1 and Fig. 5.

### EEG data collection

We used an actiCHamp amplifier and its electrodes to record a user’s scalp EEG signals. In this study, the recording electrodes were FC1, FC2, CP1, CP2, and Cz, the reference electrodes were TP9 and TP10, and the ground electrode was Fpz. The sampling frequency was 200 Hz. In the experiments, the impedance of all electrodes was kept below 10 kΩ. To reduce the interference induced by the electromyography signal and electrooculogram signal, we used a 0.5 Hz-to-50 Hz bandpass filter to preprocess the recorded raw EEG signals.

### EEG signal processing

When the user turned on the CVAR-BCW, our EEG amplifier started recording EEG signals. Obvious P300 component could only be recorded after visual stimuli appeared for several hundred milliseconds. In our study, we would read an EEG signal for each option after stimuli appeared (700 ms long). Since the sampling frequency used in our study was 200 Hz, each signal included 140 sample points. To reduce the data size, we used a downsampling filter with a sampling rate of one tenth to filter each signal. The signal after downsampling included 14 points. As described in the Experimental Procedures section, there were four flashes on each option. Therefore, the total signal of each option included 14 × 4, i.e., 56 sample points. The stepwise linear discriminant analysis algorithm is often used for P300 feature extraction [53, 54]. The principle is to multiply the collected EEG signal by an optimal weight matrix. The larger the product is, the more obvious the P300 features contained in the EEG signal. In this study, we used the optimal weight matrix $W$ obtained in the offline experiment to multiply the 56 sample points of each option. For each option, we could obtain a product. Finally, we used the bubble sort algorithm to sort the products, and the option with the largest product was the decoding result:

1 $${\mathrm{S}}_{\mathrm{i}}=\sum_{\mathrm{k}=1}^{\mathrm{N}}{\mathrm{WX}}_{\mathrm{ik}}.$$

Here, $i$ is the ${i}^{\mathrm{th}}$ option. $N$ is the number of flashes required to decode one target. For the offline experiment and online experiment, N was four, but for the optimization experiment, *N* was not constant and varied from two to eight. $W$ is the optimal weight matrix. ${X}_{\mathrm{ik}}$ is the EEG signal recorded for the ${i}^{\mathrm{th}}$ option in the ${k}^{\mathrm{th}}$ flash, and ${S}_{\mathrm{i}}$ is the product of the ${i}^{\mathrm{th}}$ option.

### Performance metrics

Relevant studies typically use the accuracy and ITR to measure the performance of a BCW [35, 55]. In this study, we also used the accuracy and ITR as performance metrics. Different from traditional BCIs, in our study, the accuracy of the CVAR-BCW was defined as the probability that the CVAR-BCW correctly reached a target. In other words, the CVAR-BCW not only needed to correctly decode the target but also needed to correctly reach it. For example, if a subject wanted to reach a chair, the BCW should successfully decode the target chair and then reach the chair and could not collide with any obstacles. In the online experiment, each subject was asked to successfully reach ten targets. We counted the total number of commands sent by each subject. For each subject, the total number divided by ten was the accuracy of the CVAR-BCW. Using the accuracy, we calculated the corresponding ITR.

Equations (2) and (3) illustrate how to calculate ITR. *N* is the number of options in the user interface, which was 6 in our study. P is the accuracy, and *T* is the time required to decode the current target [35, 55]. S is a stimulation parameter, which was 400 ms in our experiment. *R* is the number of flashes required to select the current target. For the offline and online experiments, *R* was 4, but for the optimization experiment, *R* was not constant and varied from 2 to 8. *I* is the interval between two selections:

2 $$\mathrm{ITR}=\frac{\left({\mathrm{log}}_{2}\mathrm{N}+{\mathrm{Plog}}_{2}\mathrm{P}+\left(1-\mathrm{P}\right){\mathrm{log}}_{2}\left(\frac{1-\mathrm{P}}{\mathrm{N}-1}\right)\right)}{\mathrm{T}},$$

3 $$\mathrm{T}=(\mathrm{S}*\mathrm{R}+\mathrm{I})/60.$$

In addition, we recorded the total time and total number of commands spent by each subject to reach the ten designated targets. The total time divided by ten was the average time required to reach one target. The total number of commands divided by ten was the average number of commands required to reach one target.

We also wanted to test the advantages and disadvantages of combining CV and AR with BCWs. In the automatic mode, we recorded the number of times that our CVAR-BCW did not reach targets and classified the errors using the following six types: (1) collision, i.e., collided with obstacles; (2) navigation error (the distance to the target was greater than 0.5 m, or collided with the target); (3) HMD error, e.g., the HMD did not display a complete image; (4) BCI decoding error; (5) CV error, e.g., the CV module did not find targets, and (6) others, e.g., the subject did not watch the target. The percentage of each error type was the number of each error type divided by the total number of all errors. For example, if the number of navigation errors was three and the total number of errors was ten, then the percentage of navigation error was 30%. Most errors encountered in the semiautomatic mode were related to BCI decoding, so we did not analyze the errors in semiautomatic mode.

## Data Availability

All data generated or analyzed during this study can be obtained by emailing the author (K. Liu; 2314825269@qq.com).
